# From Floral Induction to Blooming: The Molecular Mysteries of Flowering in Woody Plants

**DOI:** 10.3390/ijms231810959

**Published:** 2022-09-19

**Authors:** Liyong Sun, Tangjie Nie, Yao Chen, Zengfang Yin

**Affiliations:** 1 Co-Innovation Center for Sustainable Forestry in Southern China, College of Biology and the Environment, Nanjing Forestry University, Nanjing 210037, China; 2 Department of Biology, The Pennsylvania State University, University Park, State College, PA 16802, USA

**Keywords:** woody plants, flowering, floral organogenesis, regulatory mechanism

## Abstract

Flowering is a pivotal developmental process in response to the environment and determines the start of a new life cycle in plants. Woody plants usually possess a long juvenile nonflowering phase followed by an adult phase with repeated flowering cycles. The molecular mechanism underlying flowering regulation in woody plants is believed to be much more complex than that in annual herbs. In this review, we briefly describe the successive but distinct flowering processes in perennial trees, namely the vegetative phase change, the floral transition, floral organogenesis, and final blooming, and summarize in detail the most recent advances in understanding how woody plants regulate flowering through dynamic gene expression. Notably, the florigen gene *FLOWERING LOCUS T*(*FT*) and its antagonistic gene *TERMINAL FLOWER 1* (*TFL1*) seem to play a central role in various flowering transition events. Flower development in different taxa requires interactions between floral homeotic genes together with *AGL6* conferring floral organ identity. Finally, we illustrate the issues and corresponding measures of flowering regulation investigation. It is of great benefit to the future study of flowering in perennial trees.

## 1. Introduction

The transition from vegetative growth to flowering in plants is the most critical developmental event during the whole life history [1]. In annual plants such as *Arabidopsis*, the reproductive transition includes two successive but distinct stages, namely the vegetative phase change (the juvenile-to-adult vegetative transition) and the floral transition (vegetative-to-reproductive transition). Using the excellent model system *Arabidopsis* and well-developed genetic tools, studies in past decades have extensively unveiled complex genetic networks controlling flowering time. The five major genetically defined pathways refer to the photoperiod, vernalization, the gibberellin pathway, autonomous, and the aging pathway [2,3,4,5].

**Figure 1:**
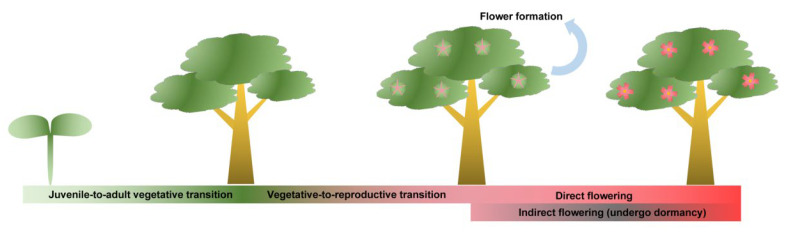
Schematic presentation of flowering phenology in woody trees. The juvenile-to-adult vegetative transition refers to a long period before woody trees first acquire flowering ability; the vegetative-to-reproductive transition commonly shows repeated cycling or rather seasonal periodicity, indicating flower formation follows the floral transition; “Direct flowering” refers to trees that finish their complete reproductive cycle during a single growing season without a dormancy period, while “indirect flowering” means the trees undergo winter dormancy before final blooming.

Annual herbaceous plants have quite short life cycles and commonly only spend several months completing the sole reproductive process. Distinctly unlike annual herbs, perennial trees usually have quite long life cycles with relatively complicated reproductive processes (Figure 1) [6]. First, they undergo a much longer juvenile phase before they first acquire flowering ability, usually lasting for several years or even decades (Table 1). Subsequently, adult woody plants show repeated cycling between vegetative and reproductive growth or rather seasonal periodicity of the floral transition. In most perennial woody plants, especially subtropical and temperate deciduous trees (apple, peach, pear, etc.), the flowering cycle spans two successive years. This means that floral induction and blooming are universally separated by a period of rest (usually characterized by the winter dormancy) [7,8,9]. These seasonal-flowering species are also called the “indirect flowering” group. In comparison, “direct flowering” species (mango, jujuba, etc.) will finish their complete reproductive cycle (i.e., the floral transition, flower bud differentiation, and blooming) during a single growing season without a dormancy period [10,11]. Even some rosaceous species (e.g., *Rosa chinensis* and *Rosa hybrida,* ‘Little White Pet’) can recurrently flower within one year [12]. What is noteworthy is that some “indirect flowering” trees can directly bloom following flower bud differentiation. For example, a portion of the floral buds of *Magnolia* × *soulangeana* ‘Changchun’ is able to skip winter dormancy and bloom in the current summer [13]. The diversity is not only exhibited in the flowering phenologic rhythm but also in flower formation in trees. Normally, the floral organogenesis process mainly involves the regulation of meristem fate and floral organ identity. All facts mentioned above imply that the regulatory mechanism may be different between annual herbaceous and perennial trees, although some flowering pathways are perhaps conserved. The genetic control of flowering time is potentially more complex in perennial plants than that in annuals and is also less understood [14]. Here, on the basis of recent research progress, we review the molecular mechanisms regulating flowering in woody plants, focusing on floral induction, floral organogenesis, and final blooming. It will enhance our understanding of how perennial trees integrate endogenous developmental processes with exogenous environments and decide whether to flower.

## 2. Molecular Regulation of Flowering Phase Transition

### 2.1. The Role of PEPB Gene Family in the Flowering Phase Transition

In angiosperms, two different clades of the *phosphatidyl ethanolamine-binding protein* (*PEBP*) gene family are well-characterized, namely *FLOWERING LOCUS T*(*FT*) and *TERMINAL FLOWER 1* (*TFL1*) [24]. They have been considered the important regulators controlling the time when woody trees become mature and are able to flower, indicating their age-dependent regulatory roles (Table 2). Therefore, *FT*, the florigen gene, is known as a floral-promoting factor. In early-flowering walnut (*Juglans regia*), which can flower at the age of 1 or 2 years old, the expression of the *FT* gene is dramatically higher than that in late-flowering seedlings [25]. Shen et al. isolated two *FT* orthologs (*PsFT1* and *PsFT2*) from *Populus simonii*, and over-expressing them in transgenic poplar clone T89 lines led to early flowering that was much earlier than the normal poplar flowering age of 8–20 years [22]. Likewise, the over-expression of *Populus deltoides FT2* shortens the juvenile phase of transgenic 717-1B4 poplar (*Populus alba* × *Populus tremula*) and controls first-time flowering [26]. On the contrary, *FT*-RNAi transgenic lines of *Jatropha curcas* show nonflowering phenotypes [27]. Evidence provided by Tränkner et al. also proves that the *MdFT1* gene is responsible for inducing flowering in juvenile plants and plays a conserved function in both annual herbaceous species (*Arabidopsis*) and perennial woody trees (apple and poplar) [28]. Interestingly, coconut palm (*Cocos nucifera*) *FT* is alternatively spliced, and the exclusive presence of the shorter *FT* variant is highly associated with the early-flowering characteristic in dwarf coconut varieties [29]. In *Liriodendron Chinense*, an *FT* alternative splice variant is specific to the *super long blooming 1* (*slb1*) mutant whose inbred offspring have much shorter juvenility (~4 months) than the wild type (usually 8–10 years) [20]. In contrast, *TFL1* contributes to the maintenance of the juvenile/vegetative phase and functions as the floral repressor. In transgenic apple (*Malus* × *domestica*) seedlings, the expression of endogenous *MdTFL1* is suppressed by its antisense RNA, thereby leading to a reduction in vegetative growth and an early-flowering phenotype [30,31]. Similar results were observed in a transgenic European pear ‘Spadona’ (*Pyrus communis*) genotype by the RNAi silencing of *PcTFL1-1* and *PcTFL1-2* [32]. Interestingly, *Apple latent spherical virus* (ALSV)-induced gene silencing of *MdTFL1* exhibits a similar strong acceleration of flowering [33]. *PopCEN1*, a member of the *CENTRORADIALIS* (*CEN*)/*TFL1* subfamily, controls shoot meristem identity, and *PopCEN1*-RNAi transgenic poplars with earlier first flowering imply its negative function in the regulation of the juvenile–adult phase transition [34]. Similarly, the CRISPR/Cas9-mediated mutagenesis of the kiwifruit (*Actinidia chinensis*) *CEN*-like genes *AcCEN4* and *AcCEN* causes rapid terminal flowering [35]. In addition, overexpressing two *TFL1* homologs of dogwood species (*Cornus* L.) in *Arabidopsis* wild-type and *tfl1* mutant results in delaying flowering time and rescuing the late flowering time phenotype, respectively [36]. All these results have demonstrated that the *TFL/CEN1* genes are functionally conserved in eudicots evolution.

In addition to the juvenile–adult phase transition, the seasonal vegetative–reproductive phase transition has also been proven to be regulated by the *FT* and *TFL1* genes (Table 2). Some *Rosa* species can flower many times within a civil year. A survey of *Rosa hybrida* screened out floral integrators (*FT*, *AP1,* and *LFY*) as postulated candidates regulating the recurrent flowering character [12]. Compared with seasonal-flowering reference plants, a loss of function or silencing of floral-repressing *TFL1-like* genes causes perpetual-flowering phenotypes in Rosaceae species, providing additional evidence that *TFL1-like* genes can also switch the transition from vegetative into reproductive growth [31,32,37]. Jones et al. [14] isolated the *FT* gene of *Eucalyptus globulus* subsp. *globulus* (Myrtaceae) and further examined its expression over a 2-year period using quantitative RT-PCR. The results demonstrate that the expression level of the *FT* homologue is temporally associated with the annual flower bud initiation (i.e., the annual transition from vegetative to reproductive growth). In adult satsuma mandarin (*Citrus unshiu*) citrus trees, a seasonal increase in the mRNA level of the citrus *FLOWERING LOCUS T* homologue *CiFT* finally stimulates floral induction [38]. Similarly, the drastically increased expression of *FT* promotes adult ‘Washington’ navel orange (*Citrus sinensis*) trees to flower [39]. Evidence from Hsu et al. has confirmed that *FT2* also plays an additional role in the initiation of seasonal flowering in poplar [26]. In Japanese pear (*Pyrus pyrifolia*), flower bud formation can be induced by manual photoperiod treatments, and far-red light at 730 nm is the most efficacious wavelength. During this process, *PpTFL1* is downregulated, while *PpFT1a* is positively correlated with flower bud formation [40]. The expression of two copies of the *MdTFL1* gene rapidly decreases during floral induction in apple, and ectopic expression delays flowering time in *Arabidopsis* [41].

As a well-known flowering activator, the *FT* gene seems not only to play an important role in floral induction but also function in (perpetual) flowering (Table 2). Meng et al. found that the distinct flowering characteristic in Chinese jujube (*Ziziphus jujuba*) is dominantly regulated by the photoperiod pathway. *ZjFT* is induced by an unregulated photoperiod and is highly expressed before flowering [11]. To a certain extent, research on the blueberry provided extra experimental evidence, namely that the over-expression of *VcFT* cloned from *Vaccinium corymbosum* in ‘Aurora’ results in the continuous occurrence of flower bud formation, flowering, and skipping the normal dormancy stage [42]. What is noteworthy is that some “indirect flowering” trees can directly bloom following flower bud differentiation. It is a very common phenomenon occurring in magnoliaceous species. For example, the *slb1* mutant of *L. Chinense* has a specific *FT* splice variant that is perhaps causal to perpetual flowering [20]. In tree peony (*Paeonia suffruticosa*), the upregulation of some flowering-related genes, such as *FT*, is associated with reblooming [43]. 

### 2.2. MicroRNAs in Flowering Phase Transition

Plant microRNAs are endogenous ~21 nt small noncoding RNA molecules that can regulate target gene expression via mRNA destabilization and translational inhibition (Aukerman and Sakai, 2003; Jones-Rhoades et al. 2006). In *Arabidopsis*, the interactions between the miR156 and miR172 family are considered to play an important role in the juvenile-to-adult vegetative phase transition via mediating the age pathway [44]. They function as inhibitors of the target *SQUAMOSA-PROMOTER BINDING PROTEIN-LIKE* (*SPL*) and *APETALA2* (*AP2*)-like genes, respectively [45]. These two evolutionarily conserved miRNAs are also responsible for the vegetative phase transition in perennial woody plants [46]. miR156 is highly expressed in young seedlings and decreases with aging in some forest species (*Acacia confusa*, *Acacia colei*, *E. globulus*, *Hedera helix*, *Quercus acutissima*, and *Populus* × *canadensis*), while the expression pattern of miR172 is completely inverse [47]. Similar results have been observed in fruit trees as well as in ornamental flowers, such as apple (*M.* × *domestica*) [48], Chinese crabapple (*Malus hupehensis*) [49], kiwifruit [50], trifoliate orange (*Poncirus trifoliata*) [51], mango (*Mangifera indica*) [52], macadamia (*Macadamia integrifolia*) [52], *Prunus mume* [53], and *Rhododendron arboreum* [54]. Autotetraploid *Lycium ruthenicum* and its diploid progenitor have late- and early-flowering characteristics, respectively, which might be caused by differential expression levels of miR156-*SPL*s and miR172-*AP2* [55]. Furthermore, the over-expression of miR156 drastically prolongs the juvenile phase in transgenic *P.* × *canadensis* [47]. Conversely, the ectopic over-expression of *JcmiR172a* from *Jatropha curcas* significantly reduces vegetative growth time in *Arabidopsis* and shortens the juvenile stage of transgenic *Jatropha* when it grows in a subtropical area [19]. Additionally, miRNAs also function in the floral transition. A multiomics analysis revealed a potential role of miRNAs in stimulating the transition from vegetative growth to reproductive growth in *M. × soulangeana* ‘Chuangchun’, in which the miR172 family and several other novel miRNAs were differentially expressed and integrated into the GA pathway [56]. In the ‘Golden Delicious’ apple tree, several miRNAs have been found to be vegetative-bud-enriched (miR156, miR159, miR398, and miR408) or floral-bud-enriched (miR164, etc.). Correspondingly, target genes, such as *SPL*s and *ARF*s, are down- or upregulated and are speculated to control the floral transition [57]. The upregulation of miRNA167h is considered to be associated with late flowering in *Prunus sibirica* via participating in the trehalose-6-phosphate (Tre6P) signaling pathway [58]. However, it is regrettable that these results obtained from bioinformatics analyses still need further experimental verification.

## 3. Regulation Mechanism of Floral Organogenesis

Flower formation is a key regulatory event from floral induction to final blooming in angiosperms, which marks the beginning of the reproductive phase of development [59]. The first developmental transition morphologically reflects that the shoot apical meristem (SAM) transits into the floral meristem (FM), which requires the activity of the *LEAFY* (*LFY*) gene [60]. Previous studies of flowering trees have shown that *LFY* homologues are highly expressed in flower buds in poplar and hickory [61,62]. Further temporal expression analyses of fruit trees (avocado, mango, peach, etc.) have demonstrated that *LFY* homologues are initiated and highly expressed in the early stages of the floral transition and floral organ differentiation [63,64,65]. The ectopic expression of *LFY* homologues from *Cedrela fissilis*, peach, and fig (*Ficus carica*) rescues the floral defect phenotype in *Arabidopsis lfy* mutants [64,66,67]. The RNAi-mediated suppression of *LFY* in *P. alba* female 6K10 clones results in floral knockdown phenotypes showing the presence of undeveloped carpels that lack stigmatic structures [68,69]. Similarly, in *JcLFY*-silenced *Jatropha curcas*, the presence of bracts and shoot buds are observed in abnormal inflorescences, and abnormal flowers are surrounded by 20 sepaloid organs [27]. These results have uncovered the crucial role of the *LFY* gene in the determination of meristem identity.

**Figure 2:**
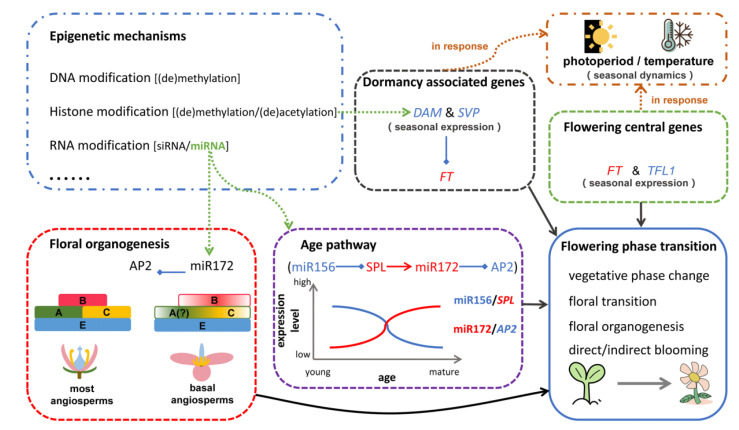
Diagram illustrating the molecular model underlying flowering regulation in perennial trees, which incorporates important/central genes and microRNAs as well as epigenetic modification. Black solid arrows indicate gene regulation; green dotted arrows indicate epigenetic modification participating in gene regulatory modules; brown dotted arrows represent responses to environmental cues.

Once SAM transits into FM, floral organ primordia arises, and the floral homeotic genes are responsible for floral organ identity during floral organogenesis. According to the interpretation of gene functions learned from floral mutants, several floral homeotic genes and miRNAs have been identified. On the basis of studies in the model plants *A. thaliana* and *Antirrhinum majus*, Coen and Meyerowitz [70] summarized and proposed the classic ABC model to explain the identity of successive floral elements. In *Arabidopsis*, these floral homeotic genes refer to *APETALA1* (*AP1*), *APETALA2* (*AP2*), *APETALA3* (*AP3*), *PISTALLATA* (*PI*), and *AGAMOUS* (*AG*). A- (*AP1* and *AP2*) and C- (*AG*) class genes control calyx and carpel formation, respectively. The interaction of A- and B- (*AP3* and *PI*) class genes is responsible for petal development, while stamens develop as a result of the synergetic activity of B- and C-class genes. A revised vision of the ‘ABC’ model, known as the ‘ABCE’ model (Figure 2), has been proposed in combination with a further understanding of *SEPALLATA* (*SEP*) gene function [71]. In addition, D-class genes, including *SEEDSTICK* (*STK*), *SHATTERPROOF1* (*SHP1*), and *SHATTERPROOF2* (*SHP2*), are sufficient to induce ovule development [72]. The genetic mechanism underlying the floral organogenesis of most angiosperm trees can be interpreted under the framework of the ‘ABC(E)’ model. Considering difficulties in establishing a genetic transformation system of woody species, the ectopic expression of floral homeotic genes in wild-type or corresponding mutants of model plants (i.e., *Arabidopsis* and tobacco) has been widely applied to investigate gene function. For example, Lemmetyinen et al. isolated two MADS-box genes from silver birch (Betula pendula), namely *BpMADS1* and *BpMADS6,* which are homologues of *AtSEP3* and *AtAG* [73]. Ectopic expression showed detective petals in both 35S::BpMADS6 transgenic *Arabidopsis* and tobacco. As expected, 35S::BpMADS1 causes global flower defects, which is consistent with its E function. Both *PI* genes from *Catalpa bungei* and *Argania spinosa* rescue petal and stamen identity when they are ectopically expressed in an *Arabidopsis pi-1* mutant [74,75]. RNAi-mediated gene silencing is another more effective and direct approach. For example, the RNAi-*AG* sweetgum trees (*Liquidambar styraciflua*) have modified inflorescence and floral morphology, with anthers and carpels converting to flat leaf-like structures [76]. The expression suppression of *AG* genes caused by RNAi finally leads to ‘carpel-inside-carpel’ phenotypes in the poplar clone 6K10 (*P. alba*) [77]. However, in the basal angiosperms and Magnoliids, floral organs are not clearly divided into different types, all of which exhibit gradual morphological transition (e.g., *Amborella trichopoda* and *Persea americana*) [78,79]. These undefined floral architectures might be attributed to a variant of the ABC model, namely ‘the fading borders model’ (Figure 2). This model is characterized by expression domains of floral identity genes that partially overlap with each other, and their expression levels are weaker in the outermost and innermost margins of the expression domains [80,81,82]. The expression patterns of floral homeotic genes determined by quantitative real-time PCR (qRT-PCR) in several *Magnolia* species provide more valid evidence. For example, *AG* is expressed in the perianth elements of *M. stellata* and shows centrifugally reduced activity [83]. In *M. grandiflora*, a weak expression of *AP3* was detected in the carpels [84]. Further gene cloning and ectopic expression experiments revealed that A/B/C-class floral homeotic genes have conserved biological functions in basal angiosperm trees and in core eudicots [85,86,87,88,89]. The *AG* gene from *M. stellata* can rescue carpel defects rather than stamen in an *Arabidopsis ag-1* mutant. Nevertheless, the ectopic expression of *AG* alternative splice variants that lack C function causes changes in the perianth elements of wild-type *Arabidopsis* [87]. Over-expressing *M. wufengensis AG* in *Arabidopsis* results in the homeotic conversion of petals into stamenoid organs, indicating its C function [85]. Both *AP3* and *PI* from *M. wufengensis* can partially rescue the loss of function in the corresponding mutant [86,88].

Interestingly, beyond floral homeotic genes, *AGL6*-like genes are identified as extra regulators involved in specifying floral organ identity [90]. Members of the ancient AGL6 subfamily of plant MADS-box genes possess diverse functions in different taxa. On one hand, *AGL6* genes perform the ‘E’ function, the same as well-characterized *SEP* genes in herbaceous plants (petunia, rice, and maize). On another hand, they are also considered to be responsible for the development of floral organs in the first whorl, which means they possess ‘A’ function, at least partially [91]. Studies in both gymnosperms and some basal angiosperms have enriched our understanding of how *AGL6* genes regulate sexual organ development in woody plants [83,92,93]. When ectopically expressing the *CpAGL6* gene from wintersweet (*Chimonanthus praecox*), transgenic plants show no ectopic floral organs but have abnormal stamen and carpel development, indicating its potential ‘E’ function [93]. Conversely, the role of the A-class gene function in *Magnolia* species can be assumed by *AGL6* (proposed by Soltis et al. [81] and further proved by Ma et al. [94] through genetic experiments). Li et al. found that the *AP1* gene from *M. wufengensis* cannot restore the sepal and petal formation of *Arabidopsis ap1* mutants, revealing the absence of A function [89]. As expected, the *M. wufengensis AGL6-2* gene can preferentially regulate tepal morphogenesis [94].

Except structural genes, miR172 has been considered to regulate floral organ identity by directly targeting the *AP2* gene. In peach (*Prunus* persica), an AP2 transcription-factor-encoding gene (*Prupe.6G242400*) was screened out as the candidate regulating single or double traits through high-resolution linkage mapping. Further study showed that a deletion spanning the miR172 binding site confers *AP2* gene miR172 resistance, resulting in double-flower formation [95]. Similarly, in kiwifruit alternative splicing leads an *AP2* transcript to lose the miR172 targeting site, thereby escaping miR172-mediated cleavage. Finally, abnormal *AP2* accumulation results in multiple perianth whorls and extended petaloid features in the mutant ‘Pukekohe dwarf’ [50]. In addition, a transposable element insertion event leads to the creation of an miR172-resistant *RcAP2-like* variant, which is highly associated with the double flower phenotype in roses (*Rosa chinensis*) [96].

## 4. *DAM/SVP* Genes Associated with Dormancy and “Indirect Flowering”

Dormancy is the representative event during the flowering cycle in “indirect flowering” deciduous trees and a survival strategy to escape the deleterious effects of winter temperatures [97]. The dormancy cycle is divided into three different stages, namely endodormancy, ecodormancy, and paradormancy [98]. Endodormancy is induced by daylight shortening and decreasing temperatures during autumn and winter and requires a certain period of chilling accumulation. Once chilling requirements have been fulfilled, floral or vegetative buds will release from endodormancy and enter ecodormancy, where the growth of buds is inhibited by adverse environmental conditions [99]. The maintenance or release of dormancy is under unique genetic control. *SHORT VEGETATIVE PHASE* (*SVP*), an *Arabidopsis* floral regulator, can represses *FT* transcription and be integrated into the thermosensory pathway [100]. Recently, the *SVP* gene and its homologue *SVP-LIKE* (*SVL*) have also been implicated in regulating flowering and growth–dormancy cycles in perennials. *35S:SVL* transgenic poplars exhibit abnormally late floral bud break to a certain degree [101]. Chromatin immunoprecipitation (ChIP) assays showed that aspen SVL binds to a CArG box on the *FT1* promoter, directly suppressing *FT1* expression [102]. In sweet cherry (*Prunus avium*), the expression pattern of *PavSVP* is closely associated with the suppression of flowering during the dormancy period. Ectopically expressing *PavSVP* in *Arabidopsis* delays flowering [103]. Kiwifruit *SVP2* has been functionally characterized as a repressor of precocious bud break during dormancy through transgenic experiments. The prolonged dormancy duration in transgenic kiwifruit can be overcome by sufficient winter chilling [104]. In addition to *SVP*s, *DORMANCY-ASSOCIATED MADS-BOX* (*DAM*) genes in rosaceous plants are central regulators of dormancy but are clustered separately in the gene tree [105]. The first report was the *evergrowing* peach mutant: the deletion of six *DAM* genes led to a complete lack of dormancy [106,107]. The biological function of *DAM*s seems distinct due to their diverse season-dependent expression patterns during the dormancy cycle (see Figures 1 and 3 in the previous review [108]). Peach (*Prunus persica*) is a good example for deeply investigating *DAM* genes. *PpeDAM1, 2, 3,* and *4* display the pattern with an approximate peak of expression during bud set. Nevertheless, the expression levels of *PpeDAM5* and *PpeDAM6* increase over the winter, suggesting they are responsible for the maintenance of bud endodormancy [109]. Additional experiments in a manually controlled environment further confirmed that the expression of *PpeDAM5* and *PpeDAM6* can be induced by photoperiod, temperature, and exogenous cyanamide [109,110]. Endodormancy release in almond cultivars is accompanied by continuously downregulated *DAM*-like expression levels [111]. In apple (*M. ×* *domestica* ‘Royal Gala’), *MdDAMb* plays a similar role in maintaining bud dormancy [8]. The RNAi-mediated repression of all *DAM* and *SVP* genes results in evergrowing apple trees with a precocious-flowering phenotype. Compared with wild-type plants, the expression of the *MdFT2* gene is elevated in the terminal buds of RNAi lines [112]. Yeast one-hybrid and dual-luciferase transient expression assays provide limited experimental evidence that DAMs can directly bind to the promoter region of the *FT2* gene in pear [113]. Quite interestingly, *VvDAM-SVPs* gene expression is regulated by *vvFT* in grapevine (*Vitis vinifera*) [114]. In addition, Zhao K. et al. found protein interactions among DAMs, and their specific expression patterns contribute to endodormancy in *P. mume* [115]. Importantly, DAM transcription factors integrate ABA signaling, GA biosynthesis, and catabolism, ultimately mediating dormancy and bud break in perennials [105].

## 5. Epigenetic Modification in Flowering Regulation

Epigenetic regulation plays an important role in plant growth and development as well as in the response to environmental stresses. The control of flowering progression, including bud dormancy release, is a complex process mediated by different types of epigenetic regulation, i.e., DNA methylation, histone modification, chromatin remodeling, and small interference RNAs (siRNAs) (Table 3) [105,116].

Cytosine DNA methylation, a stable epigenetic mark, is associated with a repressed chromatin state and the inhibition of gene expression [117,118]. During plant developmental processes, a DNA hypermethylation–hypomethylation wave often occurs in the promoter region and specifically in the bodies of active genes. In apple and tree peony (*P. suffruticosa* ‘Luhehong’), dormancy release is induced by chill conditions and accompanied by a decrease in total DNA methylation [119,120]. Additionally, apple flower bud formation is attributed to high expression levels of flowering-related genes (e.g., *SOC1*, *AP1,* and *SPL*s), which are associated with low methylation levels in the gene-body regions [121]. In the apple cultivar ‘Fuji’, dynamic patterns of DNA methylation are associated with mRNA and siRNA expression, and high CG and CHG methylation were forcefully maintained at the early stage of flower induction [122]. Similarly, in basket willow (*Salix viminalis*), the application of 5-azacytidine (5-azaC), a DNA methylation inhibitor, leads to hypomethylation in leaves at the floral transition stage and thus promotes the floral initiation and subsequent flower growth [123]. As for tree peony, 5-azaC application significantly reduces the DNA methylation level in the *PsFT* promoter region and induces higher expression of the *PsFT* gene, thereby triggering flowering [124]. In sweet cherry, Rothkegel et al. found the silencing of the *PavMADS1* and 2 genes during cold accumulation and dormancy release is related to DNA methylation and siRNAs [125]. Their recent study indicated that DNA methylation might act as an early response to low temperatures in the endodormancy period, thus regulating gene expression in a genotype-dependent manner [126]. Similar results were also observed in almond (*Prunus dulcis*) cultivars with early- and late-flowering phenotypes [127]. The DNA methylation levels in the apical buds of chestnut (*Castanea sativa*) increase and decrease during bud set and bud burst, respectively. In comparison to DNA methylation, an opposite abundance pattern of H4ac coincides with changes in bud dormancy [128], and the histone-modification-related genes *HUB2* and *GCN5L* are differentially expressed in dormant and germinating buds [129]. During the floral transition in azalea, global DNA methylation and H4ac have opposite and particular dynamics, namely increased DNA methylation levels in contrast to decreased H4ac levels [130,131,132].

Recently, more studies have uncovered the role of histone modification in dormancy and flowering regulation. Genome-wide histone modification gene families have been identified in apple, including 71 histone methyltransferases, 44 histone demethylases, 57 histone acetylases, and 26 histone deacetylases, and most of them are involved in and respond to flower induction [133]. The *PavDAM5* gene plays an important role in endodormancy maintenance in sweet cherry, and its expression level is positively related to changes over time in H3K4me3 [134]. In the Japanese pear ‘Kosui’, the reduction in active histone mark H3K4me3 contributes to the decreased expression of the *DAM* homolog *PpMADS13-1* towards endodormancy release [135]. Following the demethylation of H3K4 and the deacetylation of H3 in the region of translation start of the *DAM6* gene, H3K27me3 in the *DAM6* promoter, the coding region, and the second large intron is consistent with the repression of *DAM6,* which is responsible for dormancy release in peach [136]. Zhu et al. found multiple epigenetic events, including sRNA expression and H3K27me3 and CHH methylation, that affect the dynamic expression of *DAM* genes, thereby regulating dormancy maintenance and release in peach [137]. Therefore, the *DAM1/2/4/5* genes are significantly enriched in H3K27me3 in dormancy-released buds [138]. A study of leafy spurge (*Euphorbia esula*) revealed that a decreased enrichment of H3K27me3 and an increase in H3K4me3 were observed in the *DAM1* promoter region during endodormancy release [139]. However, the *DAM* genes are not the only target sites of histone modification. Previously, the *SVP* gene was characterized experimentally as a flowering repressor. The reduction in *AcSVP2* expression towards dormancy release in kiwifruit is attributed to a reduction in H3K4me3 and H3ac but not H3K27me3 and H3K9me3 [140]. Different from the *DAM*/*SVP* genes, the *early bud-break 1* (*EBB1*) gene encoding an APETALA2/ethylene-responsive factor (AP2/ERF) transcription factor has been identified as a positive regulator of bud break in poplar [141]. It is worth noting that the regulatory mechanism is conserved among wood species. In peach, higher levels of H3K4me3 in the 50-upstream and start codon regions of the *PpEBB* gene are associated with the induced *PpEBB* expression level, which might contribute to bud break and flowering [142]. On the contrary, the activatory mark H3K4me3 enrichment in the promoter region of *CcMADS19* is probably the cause of its higher expression, which inhibits floral induction in ‘Moncada’ mandarin (*Citrus clementina* × (*C. unshiu* × *C. nobilis*)) [143]. In addition, Fu et al. [144] proposed that bud endodormancy during chilling accumulation in peach is associated with endoplasmic reticulum stress and the unfolded protein response, which is similar to the report on *Arabidopsis* [145]. During bud dormancy release in hybrid poplar (*P. tremula × P. alba*), proteins involved in the primary metabolic pathways are differentially acetylated [146]. 

## 6. Final Remarks and Perspectives

The transition to flowering marks a key adaptive developmental switch in plants, which impacts their survival and fitness. In perennial woody plants, this process can be divided into four periods, namely the vegetative phase change, the floral transition, flower organ formation, and finally blooming. The latter three periods compose the repeated flowering cycles during the entire life. All studies summarized in this review have progressively deciphered the complex molecular mechanism of flowering regulation in perennial trees (Figure 2). Notably, the flowering phase transition (including dormancy) is controlled by an antagonistic central gene pair (*FT* and *TFL*), miR156/SPL and miR172/AP2 modules, and dormancy-associated genes in response to environmental cues. Therefore, the florigen gene, *FT*, acts as a central regulator, balancing the exogenous signaling and internal development process in a diverse flowering transition. For “indirect flowering” species, *DAM*/*SVP* genes play important roles in dormancy maintenance and release. As for flower development, floral homeotic genes, together with *AGL6* and the miR172/*AP2* module, are responsible for floral organ identity in trees. In addition, epigenetic regulation involving DNA modification, histone modification, and RNA modification widely participates in flowering events in woody species.

However, there are still some major issues that need to be addressed in the future. First, considering the complexity of woody plants, several genes may execute diverse functions at distinct tissues or developmental stages or are functional redundancies with other paralogs. That means the well-applied ectopic expression in model herbs with simple life histories may not exactly reflect gene function. Natural mutants of perennial woody plants will provide a good chance to investigate the gene function associated with flower development and flowering time regulation. Nonetheless, how to ingeniously select mutant–control pairs is still worth pondering. Except for natural mutant individuals such as *Liriodendron slb1* and *evergrowing* peach, mutational buds/branches from the original mother trees are also welcome. For example, *Magnolia* × *soulangeana* ‘Changchun’, whose floral buds can bloom in two distinct seasons, provides a compelling case for investigating dormancy and flowering as well as exploring flower development. Beyond common double-flower mutants of, e.g., peach, kiwifruit, and rose, some tree species with unisexual or polygamous flowers can be sufficient to survey their flower organ formation, such as *Woonyoungia septentrionalis*, *Osmanthus*, and persimmon (*Diospyros kaki*). However, the spontaneous mutation of gene sequences can rarely create ideal mutants with remarkable morphology differences. The reason lies in that the rate of the production of quasineutral, potentially adaptive genetic variance in quantitative characters is an order of magnitude smaller than the total mutational variance because mutations with large phenotypic effects tend to be strongly detrimental. The artificial induction of mutation through radiation (UV, X-ray, radioisotope, etc.), chemical mutagens (alkylating agent, nucleoside analog, NaN_3_, and colchicine), environment, and virus infection can accelerate mutation rates in order to generate target mutants in a very short period. This will benefit the development of forward genetic approaches in woody plants. Second, the lack of a stable regeneration system in vitro and a genetic transformation system in most woody species inhibits the development of reverse genetics and brings many more challenges in exploring specific genes related to flowering. To a great extent, plant somatic embryogenesis depends on species genotypes. Recently, using *WUSCHEL2* (*WUS2*) and *BABY BOOM* (*BBM*) gene, the morphogene-assisted transformation (MAT) has been proven to overcome genotype-dependent disorder in somatic embryogenesis in crops [147]. It might be an effective attempt to introduce this transformation system in order to make its genetic modification available in woody plants. On the other hand, a tobacco rattle virus (TRV)-dependent delivery system can help further gene editing by bypassing tissue culture [148]. Once the difficulties in stable regeneration are overcome in the era of the genome editing technology, the construction of tree early-flowering knockout or knockdown mutants using the CRISPR-Cas9 system will be expected to directly and clearly authenticate their function. Additionally, the publishing of the reference genome makes it easier to understand variation information (e.g., SNP, indel, CNV, and SV) and will also be the genetic basis of genome-wide surveys (WGBS, ChIP-seq, RIP-seq, etc.), thereby shedding light on the molecular mechanism underlying flowering regulation in woody plants.

## Figures and Tables

**Table 1:** Time of juvenile-to-adult vegetative transition in several representative woody trees.

Tree Species	Juvenile-to-Adult Vegetative Transition	Reference
*Malus* × *domestica*	4–8 years	[15]
*Camellia chrysantha*	6–8 years	[16]
*Citrus* spp.	6–10 years	[17]
*Eucalyptus globulus* ssp. *globulusis*	1–5 years	[18]
*Jatropha curcas*	5 years in subtropical areas	[19]
*Liriodendron Chinense*	8–10 years	[20]
*Populus deltoides* var. *deltoides*	5–10 years	[21]
poplar T89 (*Populus tremula* L. × *P. tremuloides*)	8–20 years	[22]
*Rosa rugosa* ‘Bao White’	>3 years	[23]

**Table 2:** Summary of *PEPB* gene family mediated flowering regulation in woody plants.

Species	Technique	Regulators	Phase Transition	References
*Actinidia chinensis*	CRISPR/Cas9	*CEN* *, *CEN4* *	Juvenile–adult phase transition	[35]
*Citrus sinensis*	qRT-PCR	*FT*	Vegetative–reproductive phase transition	[39]
*Citrus unshiu*	qRT-PCR	*FT*	Vegetative–reproductive phase transition	[38]
*Cocos nucifera*	RNA-seq	*FT*	Juvenile–adult phase transition	[29]
*Cornus* spp.	Over-expression	*TFL1* *	Juvenile–adult phase transition	[36]
*Eucalyptus globulus*	qRT-PCR	*FT*	Vegetative–reproductive phase transition	[14]
*Fragaria vesca*	Phenotyping	*KSN*	Vegetative–reproductive phase transition	[37]
*Jatropha curcas*	RNAi	*FT* *	Flowering transition	[27]
*Juglans regia*	RNA-seq	*FT*	Juvenile–adult phase transition	[25]
*Liriodendron Chinense*	RNA-seq	*FT*	Juvenile–adult phase transition; Perpetual flowering	[20]
*Malus × domestica*	Antisense expression	*FT1* *	Juvenile–adult phase transition	[30]
	VIGS	*TFL1* *	Juvenile–adult phase transition	[33]
	qRT-PCR; Over-expression	*TFL1-1* *, *TFL1-2* *	Vegetative–reproductive phase transition	[41]
*Malus × domestica* ‘Pinova’	Over-expression;	*FT1* *	Juvenile–adult phase transition	[28]
	Antisense expression	*FT1* *	Juvenile–adult phase transition; Vegetative–reproductive phase transition	[31]
*Paeonia suffruticosa*	RNA-seq; qRT-PCR	*FT*	Reblooming	[43]
*Populus deltoides*	RT-PCR; Over-expression	*FT2* *	Juvenile–adult phase transition; Vegetative–reproductive phase transition	[26]
*Populus simonii*	Over-expression	FT1*, *FT2* *	Juvenile–adult phase transition	[22]
*Populus trichocarpa*	qRT-PCR; RNAi; Over-expression	*CEN1* *	Juvenile–adult phase transition	[34]
*Pyrus communis*	RNAi	*TFL1-1* *, *TFL1-2* *	Juvenile–adult phase transition; Vegetative–reproductive phase transition	[32]
*Pyrus pyrifolia*	qRT-PCR	*FT1*; *TFL1*	Vegetative–reproductive phase transition	[40]
*Rosa* spp.	(q)RT-PCR; Phenotyping	*KSN*	Vegetative–reproductive phase transition	[37]
*Rosa hybrida*	(q)RT-PCR	*FT*	Vegetative–reproductive phase transition	[12]
*Vaccinium corymbosum*	Over-expression	*FT* *	Perpetual flowering	[42]
*Ziziphus jujuba*	(q)RT-PCR	*FT*	Direct flowering	[11]

* Gene function has been verified via genetics experiments.

**Table 3:** Epigenetic regulation of flowering in woody angiosperms.

Species	Epigenetic Modification	Modified Site	Developmental Transition	Reference
*Actinidia chinensis*	H3K4me3; H3ac	*AcSVP2*	Dormancy release	[140]
*Rhododendron* spp.	DNA methylation; H4ac	—	Floral transition	[130,131,132]
*Castanea sativa*	DNA methylation; Histone modification; H4ac	—	Bud dormancy	[128,129]
*Citrus* (‘Moncada’ mandarin)	H3K4me3	*CcMADS19* locus	Floral induction	[143]
*Euphorbia esula*	H3K4me3; H3K27me3	*DAM1*	Endodormancy release	[139]
*Malus ×domestica*	DNA methylation	—	Dormancy release	[119]
	DNA methylation	*SOC1*, *AP1*, *SPL*s, etc.	Floral transition	[121]
	Histone methylation/acetylation	—	Flower induction	[133]
*Paeonia suffruticosa*	DNA methylation	*PsFT*	Flowering	[124]
*Paeonia suffruticosa* ‘Luhehong’	DNA methylation	—	Dormancy release	[120]
*Populus tremula × Populus alba*	Lysine acetylation	Metabolic enzymes	Dormancy release	[146]
*Prunus avium*	DNA methylation; siRNAs	*PavMADS1*, *PavMADS2*	Bud dormancy and flowering	[125]
	DNA methylation	—	Endodormancy	[126]
	H3K4me3	*PavDAM5*	Bud dormancy	[134]
*Prunus dulcis*	DNA methylation	—	Dormancy release	[127]
*Prunus persica*	H3K4me3; H3K27me3; H3ac	*DAM6*	Dormancy release	[136]
	H3K27me3	*DAM1/4/5/6*	Bud dormancy release	[138]
	siRNAs; H3K27me3; CHH methylation	*DAM*s	Dormancy	[137]
	Endoplasmic reticulum stress; unfolded protein response	—	Endodormancy	[144]
*Pyrus pyrifolia* ‘Kosui’	H3K4me3	*PpMADS13-1* locus	Endodormancy phase transition	[135]
	H3K4me3	*PpEBB*	Bud break and flowering	[142]
*Salix viminalis*	DNA methylation	—	Floral transition	[123]

Note: Histone modification includes H3K4me3, H3K27me3, H3ac, and H4ac.

## Data Availability

Data sharing is not applicable to this article, as no datasets were generated or analyzed during the current study.
